# Reaching and Grasping Training Improves Functional Recovery After Chronic Cervical Spinal Cord Injury

**DOI:** 10.3389/fncel.2020.00110

**Published:** 2020-05-27

**Authors:** Chrystine Gallegos, Matthew Carey, Yiyan Zheng, Xiuquan He, Qi Lin Cao

**Affiliations:** ^1^The Vivian L. Smith Department of Neurosurgery, The University of Texas Health Science Center at Houston, Houston, TX, United States; ^2^Center for Stem Cell and Regenerative Medicine, The Brown Foundation Institute of Molecular Medicine for the Prevention of Human Diseases, The University of Texas Health Science Center at Houston, Houston, TX, United States; ^3^Summer Undergraduate Research Program, The University of Texas Health Science Center at Houston, Houston, TX, United States; ^4^Department of Anatomy and Histoembryology, School of Basic Medical Sciences, Shandong University, Jinan, China

**Keywords:** rehabilitation, hand function, chronic spinal cord injury, axonal sprouting, plasticity

## Abstract

Previous studies suggest locomotion training could be an effective non-invasive therapy after spinal cord injury (SCI) using primarily acute thoracic injuries. However, the majority of SCI patients have chronic cervical injuries. Regaining hand function could significantly increase their quality of life. In this study, we used a clinically relevant chronic cervical contusion to study the therapeutic efficacy of rehabilitation in forelimb functional recovery. Nude rats received a moderate C5 unilateral contusive injury and were then divided into two groups with or without Modified Montoya Staircase (MMS) rehabilitation. For the rehabilitation group, rats were trained 5 days a week starting at 8 weeks post-injury (PI) for 6 weeks. All rats were assessed for skilled forelimb functions with MMS test weekly and for untrained gross forelimb locomotion with grooming and horizontal ladder (HL) tests biweekly. Our results showed that MMS rehabilitation significantly increased the number of pellets taken at 13 and 14 weeks PI and the accuracy rates at 12 to 14 weeks PI. However, there were no significant differences in the grooming scores or the percentage of HL missteps at any time point. Histological analyses revealed that MMS rehabilitation significantly increased the number of serotonergic fibers and the amount of presynaptic terminals around motor neurons in the cervical ventral horns caudal to the injury and reduced glial fibrillary acidic protein (GFAP)-immunoreactive astrogliosis in spinal cords caudal to the lesion. This study shows that MMS rehabilitation can modify the injury environment, promote axonal sprouting and synaptic plasticity, and importantly, improve reaching and grasping functions in the forelimb, supporting the therapeutic potential of task-specific rehabilitation for functional recovery after chronic SCI.

## Introduction

Spinal cord injury (SCI), often caused by motor vehicle accidents, falls, violence, or sports and recreational injuries, is characterized by partial or total loss of motor and sensory functions below the level of the injury resulting in severe permanent disabilities dependent on the level and severity of the lesion (Armour et al., 2016; Loy and Bareyre, 2019). SCI patients often live with paralysis and an extremely reduced quality of life and productivity. Unfortunately, effective treatments for SCI remain elusive. Locomotor training, including conventional gait training, overground walking training, body-weight-supported treadmill training, and the recent robotic-assisted gait training, have shown great potential to promote functional recovery in SCI patients (Harkema et al., 2012; Brazg et al., 2017; Mehrholz et al., 2017; Tse et al., 2018) and thus a potential therapeutic approach for the patients with SCI. However, functional improvement by locomotion training is still limited. The current protocols for locomotion training need to be further optimized. For example, the optimal timing and extent of locomotor training remain to be determined. Importantly, the underlying neuronal mechanisms by which locomotor training promotes functional recovery are poorly understood (Cote et al., 2017). Animal studies could provide very useful information in these important aspects, which may help develop optimized locomotor training-based therapies for greater functional recovery in SCI patients.

Different locomotor training approaches have been shown to improve recovery after SCI in a variety of animal injury models (Jakeman et al., 2011; Harkema et al., 2012; Rossignol et al., 2015; Sandrow-Feinberg and Houle, 2015; Torres-Espin et al., 2018). These approaches can be categorized into voluntary and forced training. Movement in the home cage is the most common but often overlooked type of voluntary training which contributes to the spontaneous locomotor recovery after thoracic SCI (Caudle et al., 2011). Enhancing voluntary rehabilitation in an enriched environment further increases functional recovery after SCI (Lankhorst et al., 2001; Van Meeteren et al., 2003). Forced rehabilitation includes treadmill training, bicycling, or swimming (Torres-Espin et al., 2018; Loy and Bareyre, 2019). Treadmill training is the most commonly used forced training paradigm experimentally and can, with body weight assistance, also be applied to animals with severe SCI (Torres-Espin et al., 2018). Treadmill training alone promotes functional recovery after incomplete SCI (Shah et al., 2013; Jung et al., 2016) but not after complete transection (Ilha et al., 2011). However, combinatorial treatments including treadmill rehabilitation and pharmacological and electrical modulations in the lumbar spinal cord are able to partially restore locomotion in completely transected rats (van den Brand et al., 2012). Importantly, these experimental results are similar to ones from recent clinical trials for treadmill training with or without epidural simulation (Rejc et al., 2017a, b; Tse et al., 2018), suggesting that animal studies are able to provide very useful insights for designing rehabilitation treatments for SCI patients. Bicycling (Peterson et al., 2000; Bose et al., 2012) and swimming (Magnuson et al., 2009; Smith et al., 2009) are less commonly used and less effective forced rehabilitative strategies. Current rehabilitative strategies, including both voluntary and forced training, focus primarily on the recovery of hind limbs using thoracic SCI models. However, the majority of SCI patients have cervical injuries, and regaining hand functions is one of the top priorities for these patients since even partial recovery of hand functions could significantly improve the quality of their life (Anderson, 2004). More studies are much needed to investigate the therapeutic potential of rehabilitative approaches in the recovery of hand functions.

Previous studies show that task-specific training with single-pellet retrieval promotes the recovery of fine forelimb functions such as pellet reaching and grasping after cervical SCI (Girgis et al., 2007; Garcia-Alias et al., 2009; Krajacic et al., 2010). Recently, training with the Modified Montoya Staircase (MMS) has been shown to improve food pellet grasping after cervical SCI (Wang et al., 2011; Liu et al., 2017). While the detailed kinematic analysis for forelimb fine function in the MMS test is difficult to perform as in the single pellet reaching test (Farr and Whishaw, 2002; Wang et al., 2017), MMS training could provide an additional advantage for unilateral cervical SCI since the uninjured contralateral forelimb is not able to reach the pellets at the injury side. However, these studies have used either dorsal or lateral cervical laceration models, which are not relevant to the contusion SCI most commonly occurring in patients. Importantly, rehabilitation previously has been administered immediately or a few days after SCI. It remains unknown whether rehabilitation starting at chronic SCI will promote the recovery of fine forelimb function. In this study, we have attempted to fill these gaps by investigating whether rehabilitation with MMS would promote functional recovery of the forelimb after a chronic cervical contusion injury.

## Materials and Methods

All animal care, behavioral testing, and surgical interventions were performed in strict accordance to the approval of the Animal Welfare Committee at the University of Texas Health Science Center at Houston.

### Surgical Procedures

Age-matched female and male adult (3–6 months) athymic nude rats [NIH-RNU, Taconic; body weight, 190 ± 10 g (female) and 295 ± 25 g (male)] were used in this study. There were eight nude rats (five female and three male) in each group. After anesthetization performed with 4% isoflurane, rats received a dorsal laminectomy at the fifth cervical vertebral level (C5) to expose the spinal cord, and the spine was stabilized using steel stabilizers inserted under the transverse processes one vertebra above and below C5 as described in previous thoracic contusion (Walker et al., 2015). The rats were then moved to an Infinite Horizons Spinal Cord Impactor (Infinite Horizons LLC, Lexington, KY, United States) and received a 150 kdyne moderate unilateral contusive injury at the middle of the injured side spinal cord. During the injury, the spinal cord was not rotated as described previously (Lee et al., 2010, 2012). Our stabilizer was able to produce a consistent cervical contusion without rotating the spinal cord. Importantly, the injury severity shown by both histology and behavioral tests was very similar to these previous studies (Lee et al., 2010, 2012). The MMS assessment was used to determine the preferred side for the contusion injury. Afterward, the wound was sutured in layers, bacitracin ointment (Qualitest Pharmaceuticals, Huntsville, AL, United States) was applied to the wound area, 0.1 ml of gentamicin [2 mg/ml, subcutaneous (sc); ButlerSchein, Dublin, OH, United States] was injected subcutaneously, and the animals recovered on a water-circulating heating pad. Rats received analgesic agent, buprenorphine (0.05 mg/kg, sc; Reckitt Benckise, Hull, United Kingdom), twice a day for 3 days during postoperative monitoring.

### Forepaw Reach and Grasp Training and Assessments

Rats were trained in the skilled reach and grasp task with the Rat Motility Staircase (catalog #80300; Lafayette Instrument Company) for MMS assessment (Montoya et al., 1991; Klein and Dunnett, 2012). Bacon pellets (AIN-76A Rodent Tab Bacon, 45 mg; Lab Animal Supplies, Inc.) were colored per step level as previously described (Kloth et al., 2006; Klein and Dunnett, 2012; Lewandowski and Steward, 2014) using Crayola Washable Marker (Broad Line Non-Toxic Markers, Classic Colors, 10 count). Rats showed no preference for either colored or native pellets and readily ate all provided. As pellets appeared palatable, rats were only food restricted for 14–16 h prior to MMS testing days so they would not be sated for testing (∼7 g chow/rat). Food was promptly returned after testing.

The following baseline training schedule was used to determine paw preference: days 1–2, rats were introduced to 45-mg bacon pellets in their home cage to minimize neophobic response; day 3, one session of 20 min in unbaited staircase enclosure for equipment habituation; day 4–5, 1 session of 20 min each day as 5 min alone, remaining 15 min experimenter lures rat toward staircase antechamber baited with 5 pellets per well on both sides; days 6–15, one session of 15 min each day, 5 days a week. After each session, the number of pellets dropped, misplaced, eaten, and taken were tallied for each step. During this initial training, rats could access both sides of the staircase. Generally, rats consistently used one paw more successfully than the other paw over time, which was identified as the preferred paw once performance plateaued (after approximately eight normal sessions). Six rats would not perform the staircase and were excluded.

In this study, we attempted to examine the effects of the MMS training after chronic cervical SCI. We started the MMS training at 8 weeks post-injury, when rats were randomly assigned to two groups which either received MMS training or no training (control), respectively. For the MMS training group, rehabilitation was performed daily using the MMS baited only on the preferred reaching side which encouraged forced limb reaching. MMS training rats received rehabilitation for 6 weeks from 8 to 14 weeks post-injury (PI), and performed one session of 15 min each day, 5 days a week. All MMS training and control rats performed one MMS assessment of 15 min weekly by staffers who were blinded to the research groups.

After each MMS test, the number of pellets dropped, misplaced, eaten, and taken were tallied for each step, the maximum step reached, and the accuracy rate (# pellets eaten/# pellets taken × 100) were collected. The mean scores between the two groups were analyzed using repeated-measures ANOVA with the between-groups factor, Tukey’s honestly significant difference (HSD) multiple comparisons for the main effect of time, and independent samples *t*-test for the main effect of group.

### Locomotor Assessments

Rats were habituated and tested for 3 days in horizontal ladder (HL) and grooming tests prior to SCI. The HL and grooming tests were performed at 14 days PI and every other week for 14 weeks. Animals were coded, and behavioral assessments and analyses were performed by two investigators blinded to the treatment groups.

The grooming test is sensitive to sensorimotor deficits induced by cervical SCI and was performed as previously described (Bertelli and Mira, 1993; Soblosky et al., 2001). Briefly, cool tap water was applied to the animal’s head and back with soft gauze, and the animal was returned to its home cage. Grooming activity was recorded with a video camera (Sony Handycam HDR-CX440, Digital HD Video Camera Recorder) from grooming onset through at least two stereotypical grooming sequences (around 2 min), which include (1) licking of the forepaws and face washing, (2) forelimb grooming of the face, (3) repetitive licking of the body, and (4) hind paw scratching. Scoring was done according to the highest point reached by the forelimb: 0, the animal was unable to contact any part of the face or head; 1, the animal’s forepaw touched the underside of the chin and/or the mouth area, but not the nose; 2, the animal’s forepaw contacted the area between the nose and the eyes, but not the eyes; 3, the animal’s forepaw contacted the eyes and the area up to, but not including, the front of the ears; 4, the animal’s forepaw contacted the front, but not the back, of the ears; 5, the animal’s forepaw contacted the area of the head behind the ears (full range of motion). Slow motion video playback was used to score each forelimb independently.

The HL test is sensitive to locomotor deficits after cervical SCI as it requires adequate sensorimotor function to feel the ladder rungs and contact the rungs during stepping (Soblosky et al., 2001; Gensel et al., 2006). The device consists of a walkway enclosed by Plexiglas walls (10 cm tall, 100 cm long, and 7.6 cm apart) raised 15 cm above ground (height of two empty, inverted rat cages). Wooden applicator sticks (1-mm diameter) were inserted into holes drilled along the lower edge (every 0.5 cm) at every 1 cm for 1 m; rungs did not shift or rotate while in place but were still easily removed after testing. Rats were habituated to the HL apparatus in three training sessions where each session consisted of 5–10 complete transits. The ends of the runway were blocked off with red enrichment cylinders to prevent animals from escaping at the end and to encourage the animal toward the “target” end point, which was the home cage placed at the end of the walkway. The animals were free to explore and move about the apparatus, and sugared cereal was available in a goal box at the left or right side (respective to MMS paw preference). The rats were trained to travel from the start point toward the goal box without turning around and, if required, gently guided by the experimenter. A testing session consisted of two successful walks without turning or backwalking and was recorded on a video camera angled perpendicular to the rungs. The percentage of missed steps (# misstep/# total steps × 100) were calculated using slow motion video playback for each walk, and the average of both walks was the percent misstep for each week.

Baseline HL and grooming testing were performed before injury and again 2 weeks PI to ensure adequate injury deficit in locomotion. The mean HL misstep percentages and grooming scores were tallied by experimental group and plotted as a function of time PI. Score change over time was analyzed using repeated-measures ANOVA with the between-groups factor. The differences among the groups and each group over the 14 PI testing weeks were performed using Tukey HSD *post hoc t*-tests and independent-samples *t*-tests for the HL test and the Mann–Whitney *U*-test for grooming.

### Immunohistochemistry

After the last behavioral tests at 14 weeks PI, rats were anesthetized with a mixed solution of ketamine [80 mg/kg, intraperitoneal (ip)] and xylazine (10 mg/kg, ip) and perfused transcardially with 0.01 M phosphate buffered saline (PBS; pH 7.4), followed by 4% paraformaldehyde (PFA) in PBS. The injured spinal cord segments were removed, post-fixed in 4% PFA overnight, cryoprotected in 20% sucrose overnight, and 30% sucrose overnight at 4°C and embedded in OCT compound (Fisher Scientific). The cords were cryosectioned in 20-μm slices either transversely or longitudinally and mounted serially on Super Plus Gold Slides.

For eriochrome cyanine (EC) staining, slides were warmed on a hot plate for 5–10 min. Using fine-tipped forceps, the OCT embedding medium was carefully peeled away from the slide, and sections were submerged in two wells of xylene for 30 min each at room temperature (RT). Slides were then submerged in an ethanol gradient (100, 95, 70, and 50%) for 3 min each then in water for 2 min. Slides were stained with EC staining solution, placed in a differentiation solution, and dried at RT overnight. The next day, slides were placed in xylene for 10 min, and then a coverslip was mounted using Cytoseal Permount. A Zeiss Observer Z1 inverted bright field microscope was used to capture representative images at 10× resolution. Photomicrographs were assembled using Adobe Photoshop^®^ and Adobe Illustrator^®^ software.

For immunofluorescent staining, slides were blocked with 10% donkey serum in Tris buffered saline (TBS) containing 0.2% Triton X-100 (TBST) for 1 h at RT. The sections were then incubated in TBST containing 10% donkey serum and either triple-stained with polyclonal chicken anti-microtubule-associated protein 2 (MAP2; a marker for neuronal perikarya and dendrites, 1:500; Millipore), polyclonal rabbit anti-glial fibrillary acidic protein (GFAP; a marker for reactive astrocytes, 1:300; Dako), monoclonal mouse anti-rat cluster of differentiation 68 (CD68; also called Gp110 or macrosialin, a marker for macrophages and active microglia, 1:250; Chemicon); double stained with polyclonal goat anti-choline acetyltransferase (ChAT; a marker for cholinergic motor neurons, 1:50; Millipore) and polyclonal rabbit anti-synapsin 1 (syp; a marker for synaptic nerve terminals, 1:400; Synaptic Systems) or single stained with polyclonal rabbit anti-5-hydroxytryptamine (5-HT; also called serotonin, a marker for serotonergic neurons, 1:250; Immunostar) overnight at 4°C. After three washes of 5 min in PBS, sections were incubated in TBST containing 10% donkey serum, donkey anti-chicken IgY (IgG) TRITC-conjugated F(ab’)_2_ fragments (1:200; Jackson-ImmunoRes Lab, Baltimore, MD, United States), donkey anti-mouse IgG Cy5-conjugated F(ab’)_2_ fragments (1:200), donkey anti-goat IgG Rhodamine Red-X (1:200), donkey anti-rabbit IgG FITC-conjugated F(ab’)_2_ fragments (1:200), or donkey anti-rabbit TRITC-conjugated IgG Fab’ fragments (1:200) for 1 h at RT. In slides stained with MAP2-GFAP-CD68 or 5-HT primary antibodies, nuclei were stained with Hoechst 33258 (1:1,000; Anaspec) during secondary antibody incubation. For slides stained with ChAT-syp, the secondary antibody incubation was performed, washed, and then slides were incubated with DRAQ5 (1:1,000; abcam) for 15 min at RT to stain nuclei. The sections were rinsed in PBS and coverslipped with ProLong^®^ Gold antifade reagent. For the control sides, the primary antibodies were replaced with 1:200 mouse and rabbit IgGs and then followed the same staining procedure. A Zeiss Observer Z1 inverted fluorescence microscope was used to capture representative images at 20× resolution. Photomicrographs were assembled using Adobe Photoshop^®^ and Adobe Illustrator^®^ software.

### Histological Analyses

Image analysis and quantification were carried out with the observer blinded to the group assignment.

### Spared White Matter by Eriochrome Cyanine Staining

The spared white matter (WM) was determined as previously described (Cao et al., 2005, 2010). Briefly, one set of slides (every 10th section, each set containing 10 serial sections spaced 200 μm apart) was stained with EC to identify spared myelinated WM. The lesion epicenter was defined as the section containing the least amount of spared WM, with WM sparing defined as normal myelinated appearance and density (lacking cysts and degeneration). Septae or fibrous bands of tissue observed within and/or spanning areas of cystic cavitation were not considered to represent spared tissue. The total cross-sectional area of the spinal cord and the lesion boundary were measured using image analyses software (Zeiss Zen 2 Pro, blue edition). An unbiased estimation of the percentage of spared tissue was calculated using the Cavalieri method (Michel and Cruz-Orive, 1988). The percent of spared WM was then found by measuring sections every 400 μm apart for a total distance of 2,000 μm rostral and caudal to the injury epicenter and then dividing by the normalized WM area (% Spared = spared WM area/normalized WM area). The normalized WM areas were obtained from the normal spinal cord at the respective cervical segments. Mean values of percent spared area were calculated and statistically analyzed using independent *t*-tests (SPSS version 25).

### 5-Hydroxytryptamine Immunoreactivity

Images of an area of approximately 870 μm × 655 μm in the ventral horns at 0.8, 1.2, 1.6, and 2 mm caudal to the epicenter as well as in the ventral horns at least 4 mm rostral to the epicenter were captured. Image analyses software (Zeiss Zen 2 Pro, blue edition) automatically calculated the IR area by setting a threshold that was specific to fibers but excluded cell bodies. The percentages of 5-HT-immunoreactive (IR) area in caudal sections divided by the average 5-HT-IR area in three rostral sections, which are at least 4 mm away from the epicenter, were calculated in each animal. The difference in mean values in the percentage of 5-HT-IR area between groups was compared using independent *t*-tests (SPSS version 25).

### Synapsin Immunoreactivity

Quantification of synapsin + boutons around motor neurons was conducted by randomly capturing images of ventral horn at sections 1.2 and 1.6 mm caudal to the epicenter. A z-stack of 0.5-μm-thick images was captured through the depth of the section using a 63× oil objective on a Leica TCS SP5 Confocal Laser Scanning. The image slice with the strongest synapsin immunostaining was used for quantification. Five motor neurons with clear nucleus were randomly chosen for quantification. The synapsin-positive boutons around each motor neuron were outlined, and the corrected total cell fluorescence (CTCF) in the outlined area was automatically measured by ImageJ as described previously (McCloy et al., 2014). The density of synapsin-IR around each motor neuron was calculated by dividing CTCF by the outlined area, and the average synapsin-IR density in motor neurons between two groups was statistically analyzed using independent *t*-tests.

For quantification of synapsin + boutons in ventral horn, 10 image slides with strong synapsin-IR in a z-stack of 0.5-μm-thick images in a 246 μm × 246 μm frame were combined and converted to a set threshold. The total synapsin-IR areas were then calculated automatically by ImageJ using customized measurement macros and statistically analyzed using independent *t*-tests.

### Glial Fibrillary Acidic Protein Immunoreactivity, Cluster of Differentiation 68 Immunoreactivity, and Microtubule-Associated Protein 2 Immunoreactivity

GFAP-IR, CD68-IR, and MAP2-IR were used to assess the amount of astrogliosis, neuroinflammation, and spared gray matter, respectively. Hemisection images of the injured side were captured beginning at the epicenter and every 800 μm traversing rostrally and caudally using 20× resolution on a Zeiss Observer Z1 inverted microscope. GFAP-IR, CD68-IR, and MAP2-IR were analyzed using Zen 2 Pro. An outline was drawn around the lesioned hemisection, and the injury cavity was subtracted to obtain the spared spinal cord area. Within the outline, a set threshold was used to automatically calculate the areas of GFAP-IR, CD68-IR, and MAP2-IR, respectively, using customized image analysis macros in Zen software. The percentages of GFAP-IR or CD68-IR areas to the spared areas were calculated at all measured sections. Similarly, the percentages of spared MAP2-IR gray matter areas in the injury to the total gray matter areas in the uninjured contralateral side were also measured at all sections. Independent *t*-tests were used to statistically analyze the differences of average percentage of GFAP-IR, CD68-IR, or MAP2-IR areas between groups.

## Results

### Modified Montoya Staircase Training Improved Reaching and Grasping Functions After Chronic Cervical Spinal Cord Injury

**FIGURE 1:**
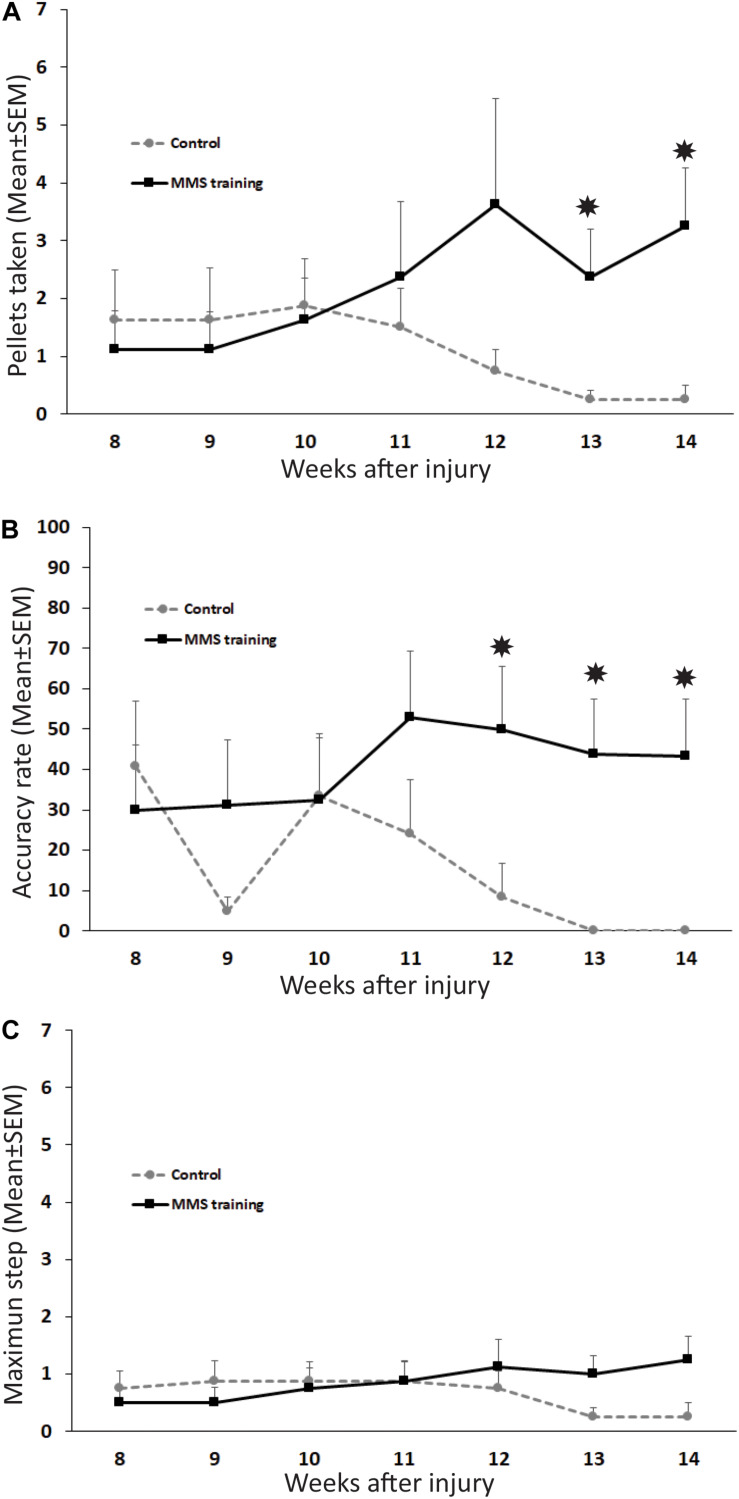
Functional recovery in reaching and grasping after Modified Montoya Staircase (MMS) rehabilitation. After unilateral cervical spinal cord injury (SCI), all rats lost the skilled forelimb functions in the MMS test in the injury side. Rats that received MMS rehabilitation weekly starting from 8 weeks after injury took significantly more pellets than the control group at 13 and 14 weeks post-injury (PI) [**(A)**, *p* = 0.037 and 0.021, respectively]. The number of pellets taken were significantly increased at 14 weeks PI compared to 8 weeks PI in the MMS rehabilitation group [**(A)**, *p* = 0.038] but not in the control group, in which the difference between the same two time points was not significant [**(A)**, *p* = 0.181]. The accuracy rate was also significantly higher in MMS rehabilitation rats compared to control ones at 12, 13, and 14 weeks PI [**(B)**, *p* = 0.039, 0.015, and 0.0195, respectively]. The accurate rate was significantly decreased in the control group at 13 and 14 weeks PI compared to 8 weeks PI [**(B)**, *p* = 0.007 and 0.009, respectively]. There were no significant differences at these later time points compared to 8 weeks PI in the MMS training group [**(B)**, *p* = 0.015 and 0.0195, respectively]. In terms of maximum step reached, there was no significant difference between the MMS rehabilitation group and the control group **(C)**. There was no significant difference from 8 weeks PI to 13 or 14 weeks PI in both groups **(C)**. Results represent mean ± standard error of the mean (SEM); *n* = 8 in each group. *P < 0.05.

To test whether MMS rehabilitation can promote functional improvement in reaching and grasping after chronic unilateral cervical contusion, the injured animals with and without rehabilitation were compared using the MMS test. The parameters assessed in this test are indicative of the respective functions in the forelimb: maximum step level for the forelimb’s range of motion, pellets taken for the activation of extensors in the forelimb, and accuracy rate for activation of forelimb flexors to carry the taken pellet accurately to the mouth and eat it. Additionally, coloring the pellets allowed us to code the steps and increase the sensitivity of testing (Kloth et al., 2006; Klein and Dunnett, 2012; Lewandowski and Steward, 2014). Normally, uninjured rats achieve successful reaching, grasping, and eating to at least step 5 and generally exhibit skilled handedness on their preferred side (Nikkhah et al., 2001). Following SCI, reaching was significantly impaired on the paw ipsilateral to the lesion, and the number of pellets taken, eaten, and range of motion of the maximum step level were significantly decreased after injury compared to baseline tests before injury. We first examined the difference of male and female rats in the MMS test before and after SCI. Our statistical analyses showed that there was no significant difference in the MMS test between female and male rats in the control or rehabilitation group before SCI or after SCI at all tested time points. Thus, results of female and male rats in each group were combined, and differences between control and rehabilitation groups were directly compared. MMS training, starting at 8 weeks after SCI for 6 weeks consecutively, increased the functional recovery of forelimb fine movement. The number of pellets taken was significantly increased at 13 and 14 weeks PI in MMS rehabilitation rats compared to untrained control rats (Figure 1A). Accuracy rate for the preferred paw in MMS training group was significantly higher compared to the control group at 12 to 14 weeks PI (Figure 1B). There was no significant difference in the maximum step reached between MMS rehabilitation and control groups (Figure 1C). The performance in the staircase test seemed improved in the MMS group but declined over time. Changes of all three tests over time in each group were further statistically analyzed. The number of pellets taken were significantly increased at 14 weeks PI compared to 8 weeks PI in MMS rehabilitation group but not in the control group, in which the difference between the same two time points was not significant. In contrast, the accuracy rate was significantly decreased in the control group at 13 and 14 weeks PI compared to 8 weeks PI, while there were no significant differences at these later time points compared to 8 weeks PI in the MMS training group. The maximum step reached in both groups was not significantly different from 8 weeks PI to 13 or 14 weeks PI. The data suggest that task-specific MMS rehabilitation could promote functional recovery in reaching and grasping of the forelimb and paw after chronic cervical SCI.

### Modified Montoya Staircase Training Did Not Increase the Untrained Locomotion After Chronic Spinal Cord Injury

**FIGURE 2:**
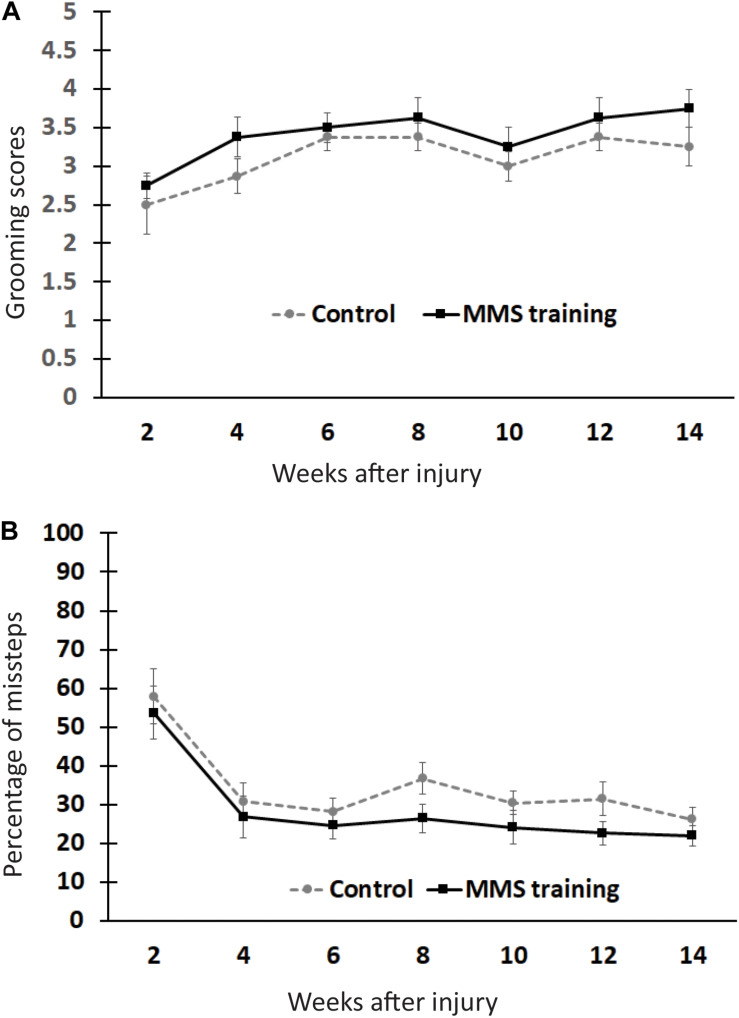
Effect of Modified Montoya Staircase (MMS) rehabilitation on untrained gross forelimb functions. After spinal cord injury (SCI), all rats lost gross forelimb function in the grooming test **(A)** and horizontal ladder (HL) test **(B)**. The spontaneous recovery in grooming scores was observed in both MMS rehabilitation and control groups from 6 to 14 weeks post-injury (PI) compared to 2 weeks PI **(A)**. However, there were no significant differences between the two groups at any tested time points **(A)**. The percentages of missteps were significantly decreased in both MMS rehabilitation and control groups from 4 to 14 weeks PI compared to 2 weeks PI **(B)**. The differences between these groups were not significant at any tested time point. Results represent mean ± standard error of the mean (SEM); *n* = 8 in each group.

To test whether MMS rehabilitation can promote recovery in untrained locomotion, injured animals with and without rehabilitation were compared using the grooming and HL tests (Bertelli and Mira, 1993; Gensel et al., 2006). The grooming score was not significantly different between MMS rehabilitation and control groups at any time point (Figure 2A). The percentage of misstep in the HL test was also not significantly different between the two groups at any tested time point (Figure 2B). These data suggest that reach–grasp training does not improve untrained gross forelimb functions.

### Modified Montoya Staircase Rehabilitation Promoted Serotonergic Fibers Sprouting

**FIGURE 3:**
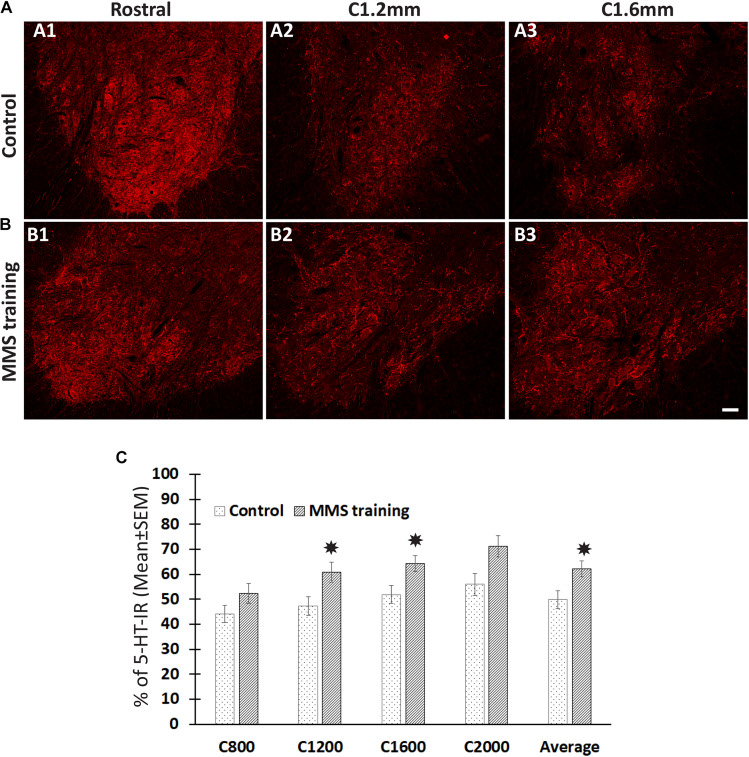
Sprouting of serotonergic axons after Modified Montoya Staircase (MMS) rehabilitation. Representative images of 5-hydroxytryptamine (5-HT)-immunoreactive (IR) fibers in the cervical ventral horns 4 mm rostral, 1.2 and 1.6 mm caudal to the injury epicenter were taken in rat without rehabilitation **(A1–A3)** or with MMS rehabilitation **(B1–B3)**. The quantification showed that MMS rehabilitation significantly increased the amount of 5-HT-IR fibers in ventral horns 1.2 and 1.6 mm caudal to the epicenter and the average from 0.8 to 2 mm caudal to the injury [**(C)**, *p* = 0.042, 0.032, and 0.039, respectively]. Results in **(C)** represent mean ± standard error of the mean (SEM); *n* = 5 in each group. Scale bar = 50 μm **(A,B)**. *P < 0.05.

The descending 5-HT-positive reticulospinal tract plays important roles in voluntary movement (Perrier and Cotel, 2015). To investigate whether MMS rehabilitation has effects in the sprouting of serotonergic fibers, the number of serotonergic fibers was identified by 5-HT immunohistochemical staining and quantified in the ventral horn, the major target area of descending serotonergic fibers in the spinal cord, at 0.8, 1.2, 1.6, and 2 mm caudal to the injury epicenter as well as 4 mm rostral to the epicenter. The quantification showed that the number of 5-HT-IR fibers in the ventral horns caudal to the injury was significantly decreased compared to ventral horns rostral to the injury for both MMS training and control groups (Figures 3A–C). The percentage of 5-HT-IR fibers in the ventral horns caudal to the injury to ones in the ventral horns rostral to injury was less than 50% at 0.8 mm caudal to the injury and gradually increased caudally away from the injury in the injury alone control group (Figures 3A–C). The same changing pattern of 5-HT-IR fibers in the ventral horns was also observed in MMS training animals. However, MMS training animals displayed very marked sprouting and had a significantly higher percentage of 5-HT-IR fibers at C1.2, C1.6, and the average of C0.8 to 2 mm caudal to the injury in comparison with the control group (Figures 3A–C). These data suggest that task-specific rehabilitation promotes the sprouting of serotonergic axons.

### The Effect of Modified Montoya Staircase Rehabilitation on Synaptic Plasticity After Chronic Spinal Cord Injury

**FIGURE 4:**
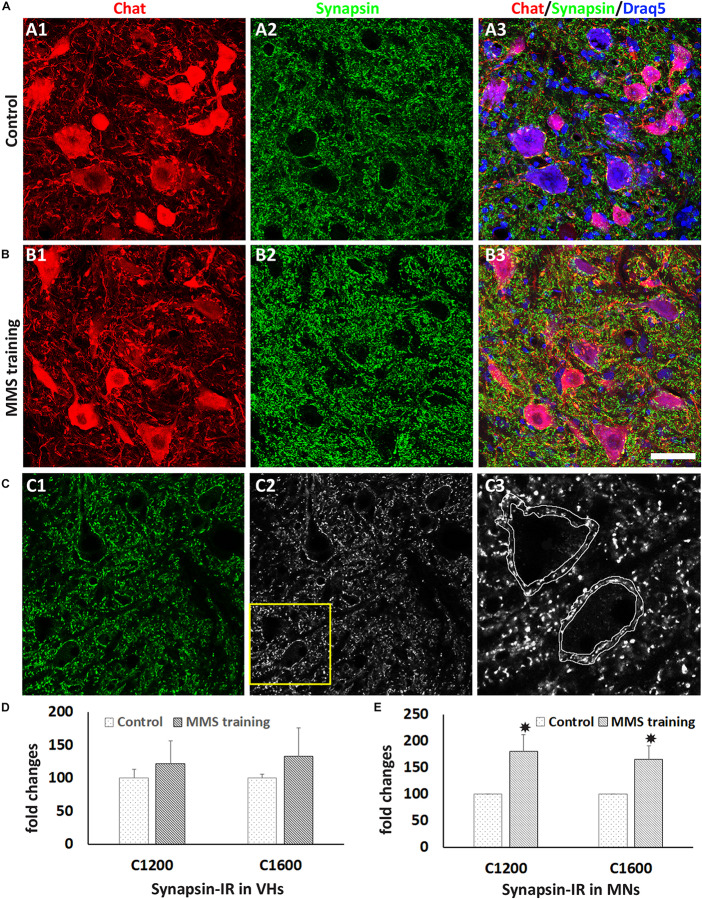
Synaptic plasticity after Modified Montoya Staircase (MMS) rehabilitation. Motor neurons and presynaptic terminals in the ventral horn were labeled immunohistochemically using antibodies against choline acetyltransferase (ChAT) and synapsin, respectively **(A,B)**. Representative immunolabeling images in **(A)** and **(B)** were taken from the cervical ventral horn at 1.2 mm caudal to the injury epicenter in the rat without rehabilitation **(A1–A3)** or with MMS rehabilitation **(B1–B3)**. Illustration of quantifying synapsin-immunoreactive (IR) terminals around motor neuron **(C)**. Original confocal image **(C1)** was converted to black–white picture **(C2)**, and synapsin + boutons around was outlined **(C3)**. The total synapsin immunoreactivity in the outlined area was automatically measured by ImageJ. The quantification showed that the total number of synapsin + synaptic terminals was not significantly different between control and MMS groups in the cervical ventral horn 1.2 or 1.6 mm or other distances caudal to the epicenter **(D)**. However, the number of synapsin + synaptic terminals around ChAT + motor neurons in the cervical ventral horn 1.2 or 1.6 mm caudal to the epicenter was significantly increased in MMS rehabilitation rats compared to control ones [**(E)**, *p* = 0.041 and 0.033, respectively]. Results in **(D,E)** represent mean ± standard error of the mean (SEM); *n* = 5 in each group. Scale bar = 50 μm **(A,B)**. *P < 0.05.

Synaptic plasticity plays important roles in functional recovery after SCI (Liu et al., 2015). To examine the effects of MMS rehabilitation in synaptic plasticity, the synapsin expression around ventral horn neurons located in the spinal cord caudal to the lesion site was examined in both MMS-trained and untrained groups using an immunohistochemical staining method. Synaptic terminals around motor neurons and other neurons in the ventral horn can be identified by synapsin immunostaining (Figures 4A,B). The overall synapsin expression in the ventral horn had a trend to increase in the MMS training group compared to the control group (Figures 4A–C), but the differences between the two groups were not statistically significant (Figure 4C). However, the amount of synapsin-IR synaptic terminals around the motor neurons in ventral horns was significantly greater in the MMS training group compared to the control group at 1.2 and 1.6 mm caudal to the epicenter (Figures 4A,B,D). The data suggest that MMS training promotes the presynaptic plasticity of spinal cord motor neurons caudal to the injury.

### Effects of Modified Montoya Staircase Rehabilitation in Astrogliosis and Neuroinflammation After Chronic Spinal Cord Injury

**FIGURE 5:**
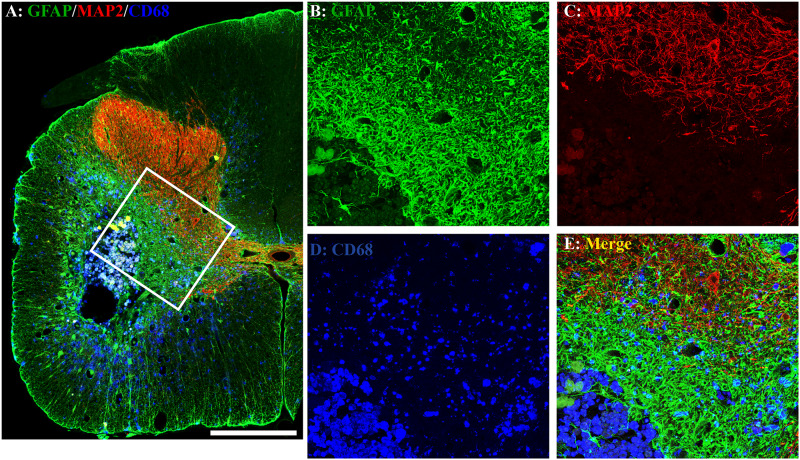
Astrogliosis and neuroinflammation after chronic spinal cord injury (SCI). Astrogliosis, neuroinflammation, or spared gray matter were evaluated by an immunohistochemical staining method using antibody against glial fibrillary acidic protein (GFAP), an astrocyte-specific marker **(A,B)**, cluster of differentiation 68 (CD68), a marker for macrophage and active microglia **(A,D)**, or microtubule-associated protein 2 (MAP2), a neuronal specific marker **(A,C)**, respectively. In the injury area, the unilateral contusion resulted in the formation of a cavity, which was surrounded by an astroglial scar. The hypertrophied CD68 + macrophages were aggregated around the lesion border and scattered in the spared gray and white matters near the injury. A representative triple-immunostaining image was taken from the spinal cord 0.8 mm caudal to the epicenter of a Modified Montoya Staircase (MMS) rehabilitation rat **(A)**. The higher magnification images of the defined area [**(A)**, white box] were shown in **(B–E)** for GFAP-IR, MAP2-IR, CD68-IR, or merged, respectively. Scale bar = 200 μm in **(A)**.

**FIGURE 6:**
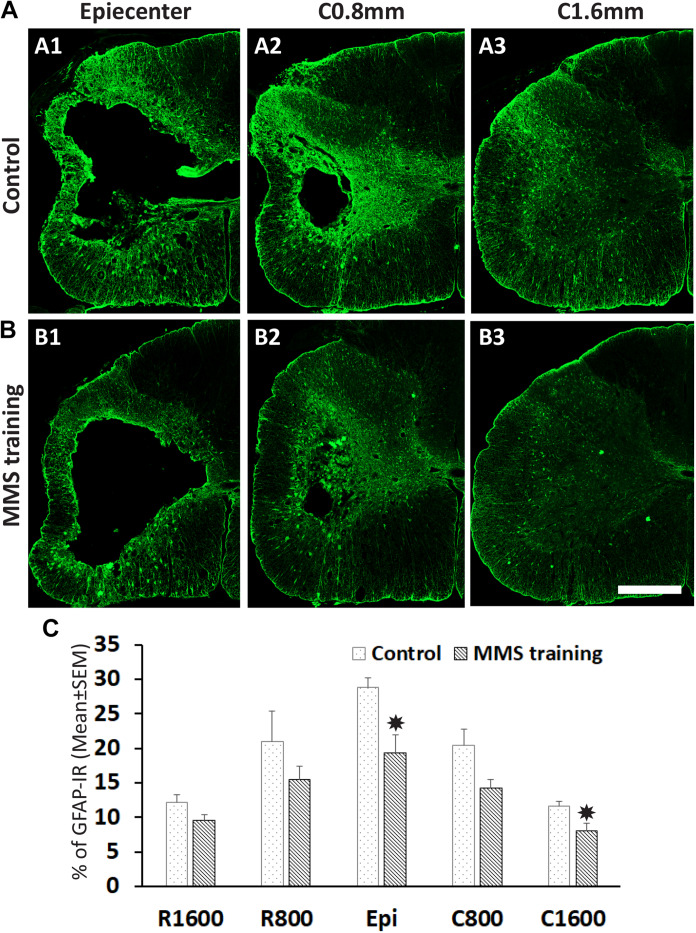
Effect on astrogliosis after Modified Montoya Staircase (MMS) rehabilitation. Representative glial fibrillary acidic protein (GFAP)-immunoreactive (IR) images of spinal cord at epicenter, 0.8 and 1.6 mm caudal to the injury epicenter were taken from the rat without rehabilitation **(A1–A3)** or with MMS rehabilitation **(B1–B3)**. The quantification showed that MMS rehabilitation significantly decreased the percentages of GFAP-IR areas to total spared areas at the epicenter and 1.6 mm caudal to the epicenter [**(C)**, *p* = 0.011 and 0.032, respectively]. Results in **(C)** represent mean ± standard error of the mean (SEM); *n* = 5 in each group. Scale bar = 200 μm **(A,B)**. *P < 0.05.

**FIGURE 7:**
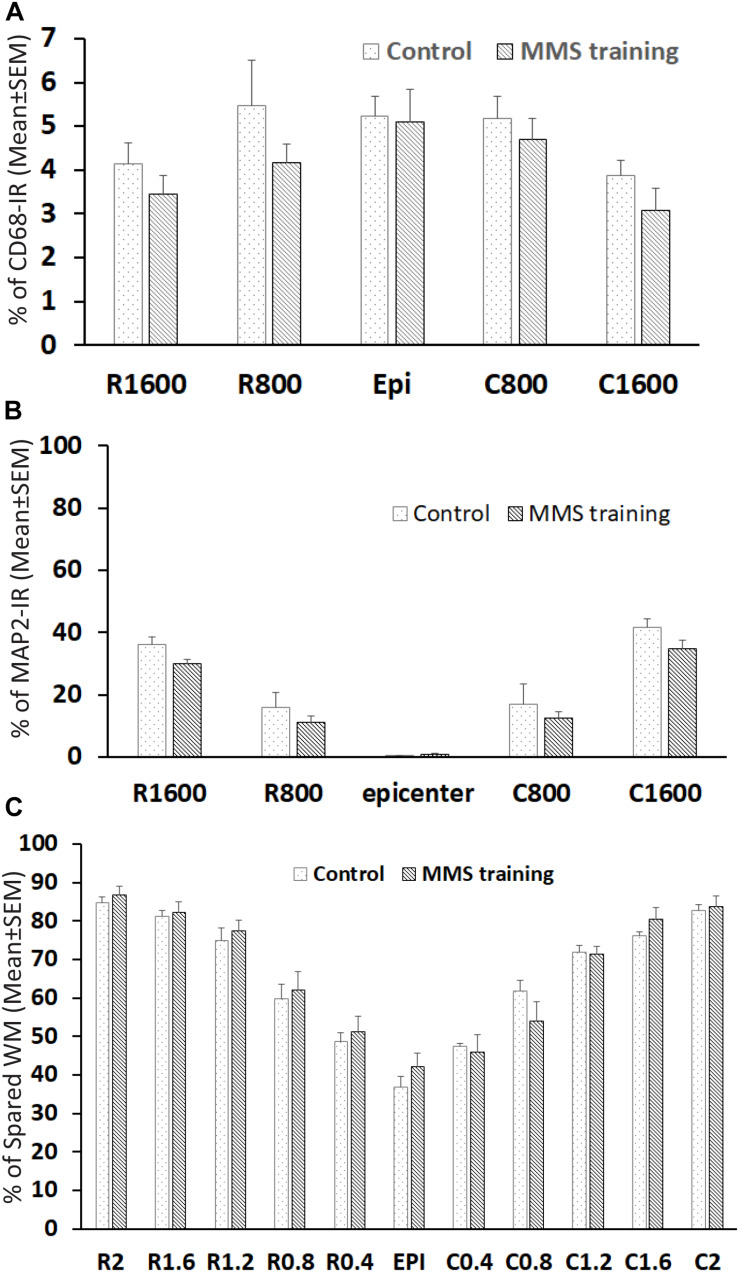
Effect on neuroinflammation and spared white and gray matters after Modified Montoya Staircase (MMS) rehabilitation. The neuroinflammation and spared gray matter were quantified with an immunohistochemical staining method using antibody against CD68 and MAP2, respectively. The spared white matters (WMs) were quantified using eriochrome cyanine (EC) staining. The quantification showed no significant difference between control and MMS rehabilitation groups in percentages of CD68-IR **(A)**, percentages of spared gray matters **(B)**, or percentages of WMs **(C)**. Results in **(A–C)** represent mean ± standard error of the mean (SEM); *n* = 5 in each group.

The unilateral contusion in nude rats resulted in the formation of a cavity, which extended from the epicenter both rostrally and caudally, and the activation of astrocytes, which formed the astroglial scar around the cavity (Figure 5). Importantly, this injury caused robust neuroinflammation, which persisted at the chronic stage with the hypertrophied CD68 + macrophages aggregated around the lesion border and scattered in the spared gray and white matters near the injury (Figure 5). These pathophysiological changes in nude rats are comparable to ones observed in non-immunodeficient rats after a similar injury (Soblosky et al., 2001; Gensel et al., 2006). To examine the effects of MMS training in astrogliosis, we measured the expression of GFAP, an astrocyte-specific marker, in the injured spinal cord using an immunohistochemical staining method (Figures 6A,B). The percentages of GFAP-IR areas in the spared spinal cord were quantified. The quantification data showed that the expression of GFAP was significantly decreased in the MMS group compared to the control group at the epicenter and 1.6 mm caudal to the epicenter (Figure 6C). These data suggest that MMS rehabilitation decreases astrogliosis in the injured spinal cord. To examine the effects of MMS rehabilitation in neuroinflammation, we measured the expression of CD68, a marker for macrophage and active microglia, in the injured spinal cord using an immunohistochemical staining method. The quantification showed that the amount of CD68-IR cells in the injured spinal cord was not significantly different between MMS rehabilitation and control groups in any examined spinal cords rostral and caudal to the injury epicenter, although there was a general trend to decrease for MMS rehabilitation rats (Figure 7A). These data suggest that MMS does not significantly affect neuroinflammation after chronic SCI.

### Effects of Modified Montoya Staircase Rehabilitation in the Spared White and Gray Matters After Chronic Spinal Cord Injury

The effects of MMS rehabilitation on residual white and gray matter were assessed using EC staining and MAP2 immunofluorescence, respectively. The myelination in WM can be identified by EC-dark blue staining. MAP2 is expressed in the cytoskeleton of neurons and their processes, and MAP2-IR staining easily defines the gray matter in the spinal cord. The unilateral contusion resulted in maximum loss of both gray and white matters at the injured epicenter, where almost all gray matter is lost with minimal spared WM surrounding the cavity. The spared white and gray matters gradually increased with increasing distances from the lesion epicenter rostrally and caudally (Figures 7B,C). Quantification showed that there was no significant difference between MMS and control groups in spared WMs by EC staining (Figure 7B) or in spared gray matters by MAP-IR staining (Figure 7C). These data suggest that MMS rehabilitation did not change the injury size, as far as spared white and gray matters were concerned.

## Discussion

In this study, we tested whether MMS rehabilitation will promote the recovery of fine forelimb function after chronic cervical SCI. Our results showed that task-specific training with MMS promoted the recovery of food pellet reaching and grasping but without functional gain in untrained tasks, shown by HL walking and grooming, after chronic cervical contusion. Our results further showed that rehabilitation improved anatomical outcomes after chronic cervical SCI such as enhanced sprouting of descending 5-HT + axons, increased numbers of synapses around motor neurons in the cervical ventral horn caudal to the injury, and decreased astrogliosis in the spinal cord caudal to the lesion.

### Task-Specific Recovery by Modified Montoya Staircase Rehabilitation After Chronic Cervical Spinal Cord Injury

Task-specific rehabilitation using the single pellet grasping task (Girgis et al., 2007; Krajacic et al., 2009) or MMS (Garcia-Alias et al., 2009; Liu et al., 2017) has been shown to promote the recovery of reaching and grasping after acute cervical SCI, but rehabilitation begins a few days after SCI in these studies. However, it is impractical clinically to start rehabilitation after acute SCI due to the frequent occurrence of multiple injuries in patients with SCI. The spinal shock shortly after injury, such as the transiently blocked transmission of spared axonal pathways (Hiersemenzel et al., 2000) and the reduced excitation in the spinal cord caudal to the lesion (Harvey et al., 2006; Chen et al., 2018), could also make rehabilitation impossible after acute SCI. Furthermore, the majority of SCI patients have chronic cervical injuries. It is critical to develop effective therapies to promote the recovery of hand function in these patients and thus to significantly improve the quality of their lives. Importantly, the repair of chronic SCI faces unique challenges which do not exist for treatments of acute or subacute SCI, such as the formation of the cavity (Ramer et al., 2014; Courtine and Sofroniew, 2019) and the established and dense glial scar around the injury. These changes represent additional barriers to repair chronic SCI. Many therapeutic interventions are effective in acute or subacute SCI but not in the chronic setting, such as delivery of neurotrophin factors (Shumsky et al., 2003; Tobias et al., 2003; Chen et al., 2006), transplantation of neural stem cells (Nishimura et al., 2013), or peripheral nerve grafting (DePaul et al., 2017). Thus, it is important to examine the therapeutic potential of task-specific rehabilitation for the recovery of fine forelimb function after chronic SCI. A previous study shows that delayed initiation of MMS rehabilitation at 1 month after injury of the cervical dorsal column promotes the recovery of fine forelimb functions (Wang et al., 2011), and combination of rehabilitation with local delivery of cABCase further promotes greater functional recovery. In this study, we started MMS rehabilitation at 2 months after cervical SCI. Our results showed that rehabilitation increased the number of pellets taken and the accuracy rate in the MMS test, indicating that MMS rehabilitation is able to improve function of food pellet reaching and grasping after chronic cervical SCI. The present study extended the effective treatment window of task-specific rehabilitation to the chronic stage, indicating that rehabilitation could be a potential effective therapy for patients with chronic SCI. Our results further show that functional benefits by MMS rehabilitation could be due to both promote functional gain (such as the pellets taken) and prevent the functional loss (such the accurate rate). It is very interesting to note the trend of drop in performance in the staircase test in the control group over time, suggesting that low-intensity and reduced frequency of training like weekly testing may not be sufficient for functional improvement or could even be detrimental. These results highlight the importance of MMS training intensity and frequency in the recovery of fine forelimb function after SCI.

### Effects of Modified Montoya Staircase Rehabilitation in Untrained Tasks After Chronic Cervical Spinal Cord Injury

Our results showed that task-specific training with MMS failed to result in functional gain in untrained tasks, shown by HL walking and grooming, after chronic cervical contusion. These results are consistent with previous studies showing task-specific functional recovery of rehabilitation following SCI. For example, step training in spinally transected cats has produced improvements in locomotion but not in standing (Lovely et al., 1986, 1990; Edgerton et al., 1997). Conversely, a specific stand-training regimen in those spinalized cats has improved standing but not locomotor ability (De Leon et al., 1998; Edgerton et al., 2008). Similarly, complete spinal rats trained to stand have shown poorer ability to perform an untrained motor task that involved lower limb flexion as compared with step-trained and non-trained rats (Bigbee et al., 2007). Injured rats trained for skilled reaching have improved reaching ability, but make more missteps than untrained rats when walking along an HL, and general environmental enrichment extinguishes paw reaching (Girgis et al., 2007; Garcia-Alias et al., 2009; Krajacic et al., 2009). In SCI patients, stepping rehabilitation has improved their ability to step but worsened their ability to stand. In contrast, stand training has increased their ability to stand but not to step (Rejc et al., 2017a, b). These studies indicate that intensive rehabilitation of one task may improve performance of that task at the expense of others which are not trained. In the present study, however, rehabilitation of skilled paw function with MMS after chronic cervical SCI did not produce deficits in other untrained motor tasks, such as HL and grooming. A previous study has shown that rehabilitative training with single pellet reaching and grasping initiated 4 days after a cervical dorsolateral quadrant lesion in adult rats promotes plasticity and task-specific recovery but also results in impairment in the untrained task (Girgis et al., 2007). However, delayed onset of the same reaching training to 12 days after the same injury improves fine reaching skill of the forelimb without negative effects in untrained task (Krajacic et al., 2009). Similarly, after a C4 dorsal funiculus cut, skilled grasping training with MMS initiated at 4 days after injury had negative effects in untrained ladder walking (Garcia-Alias et al., 2009). But delaying the training to 1 month after injury slightly improves performance in ladder walking (Wang et al., 2011). These studies suggest that the onset of rehabilitation after SCI plays a critical role in functional recovery. Time-dependent effects of locomotion training are also found in the hind limbs after thoracic SCI. In spite of the general assumption that starting rehabilitation sooner rather than later after SCI increases chances for functional improvements (Brown et al., 2011; Popok et al., 2017; Torres-Espin et al., 2018), rehabilitation initiated acutely after SCI, such as locomotion training (Li et al., 2013; Marques et al., 2018; Ung et al., 2010) or swimming (Smith et al., 2009), has negative effects on locomotor function. Delayed onset of locomotion training promotes the recovery of locomotion (Marques et al., 2018). The underlying mechanisms of how the onset of rehabilitation affects functional recovery after SCI remains poorly understood. However, competition and interaction between tasks do not occur after rehabilitation in chronic SCI, suggesting that it is possible to combine multiple task-specific trainings or task-specific training with broader training approaches such as an enriched environment at the chronic stage. It will be interesting to investigate the therapeutic potential of these combinatorial rehabilitative approaches for chronic SCI in the future.

### Axonal Sprouting and Synaptic Plasticity by Modified Montoya Staircase Rehabilitation After Chronic Cervical Spinal Cord Injury

In addition to functional benefits, MMS training has resulted in several anatomical improvements in the spinal cord below the injury, such as the sprouting of serotonergic fibers, the increased synaptic densities in motor neurons, and the reduced astrogliosis. Our results show that the amount of serotonin fibers in the ventral horns caudal to the injury is significantly increased in the MMS rehabilitation group compared to the control group, which is consistent with previous studies showing that rehabilitation can promote plasticity of serotonergic axons after SCI (Liu et al., 2017). The descending serotonergic pathway plays important roles in initiating and modulating locomotion (Berger and Takahashi, 1990; Ghosh and Pearse, 2014; Baker et al., 2015). It may also play important roles in mediating functional recovery of the hands after SCI (Baker, 2011; Baker and Perez, 2017). The roles of the descending corticospinal tract (CST) in reaching and grasping of forelimbs have been well recognized (Whishaw et al., 1998; Wang et al., 2017). The sprouting of lesioned or spared CST has been correlated with functional recovery induced by a variety of treatments including rehabilitative training (Park et al., 2008; Wang et al., 2011; Lewandowski and Steward, 2014; Liu et al., 2017). However, a previous study has shown that rats trained to grasp food pellets following cervical SCI have a significantly higher success rate than untrained rats even after pyramidotomy, suggesting that CST sprouting is not the only mechanism to promote functional recovery following rehabilitation training (Krajacic et al., 2010). A recent study has elegantly demonstrated that cortical spinal neurons in the cortex make new connections with neurons of the reticulospinal tract (RtST) to form a cortico-reticulospinal detour circuit after SCI, and the spared serotonergic RtST serves as a relay circuit of injured CST functional recovery of motor function (Asboth et al., 2018). Interestingly, this circuit reorganization only occurs after rehabilitation and neuromodulation, and recovered functional improvements remain even after terminating training and neuromodulation, suggesting a persistent exercise-induced reorganization of locomotor circuits (Asboth et al., 2018). The increased serotonergic fibers in the ventral horns below the injury in this study could partially contribute to the functional recovery of forelimb reaching and grasping by MMS training after chronic SCI.

Besides the sprouting of serotonergic fibers, another anatomical improvement by MMS rehabilitation is the increase in synaptic plasticity. Previous studies have shown that treadmill training increases the expression of synaptophysin and the number of synapses around ventral motor neurons in the lumbar spinal cord after thoracic SCI, and importantly, this was associated with an improved ability to perform stepping (Ying et al., 2005; Petruska et al., 2007; Goldshmit et al., 2008; Wang et al., 2015). Consistent with these studies, our results showed that the expression of synapsin, an important presynaptic protein, was significantly greater around ventral horn motor neurons in the cervical spinal cord caudal to the lesion in the MMS rehabilitation group compared to the control group (Figure 4), suggesting that MMS rehabilitation increases the amount of synaptic inputs onto cervical motor neurons and such enhanced synaptic connections could contribute to MMS training-induced functional recovery after chronic SCI. Previous studies show that formation of synapses is activity dependent (Petruska et al., 2007; Ichiyama et al., 2008). MMS or treadmill training may stimulate activity within neural circuits that control locomotion, and with repeated training sessions, synaptic connections within the circuitry could be reinforced and strengthened. Rehabilitation may promote the formation of new synapses connecting the ventral motor neurons and the sprouting axons, including the sprouting serotonergic fibers in this study. Rehabilitation may also prevent synaptic stripping in motor neurons after SCI. Previous studies show that SCI causes profound dendritic atrophy and synaptic stripping in the motor neurons caudal to the lesion, which are not directly injured by the trauma (Liu et al., 2014; Wang et al., 2015). Importantly, treadmill training is able to prevent these degenerative changes in motor neurons (Wang et al., 2015). These two mechanisms may contribute to the increased synaptic inputs to cervical motor neurons induced by MMS rehabilitation.

Glial scar formation is a reactive cellular process involving astrogliosis that inhibits axonal growth following SCI (Silver and Miller, 2004; Yiu and He, 2006; Tran et al., 2018). The reactive astrocytes and their deposition of extracellular matrix molecules, such as chondroitin sulfate proteoglycans, serve as the major physical and chemical barriers for axonal regeneration (Silver and Miller, 2004; Yiu and He, 2006) and synaptic plasticity (Massey et al., 2006). Our result showed that astrogliosis was significantly decreased in the epicenter and in the spinal cords caudal to the epicenter in the MMS rehabilitation group compared to the control group. These data suggest that MMS rehabilitation can provide a more favorable microenvironment that could promote axonal sprouting and synaptic plasticity, which in turn contribute to functional recovery by rehabilitation. Our results showed that MMS rehabilitation did not change the neuroinflammation and the spared white and gray matters around the injury. These data suggest that MMS rehabilitation does not change the injury size, highlighting the importance of enhanced axonal and synaptic plasticity for functional improvement by rehabilitation following chronic SCI.

### Combining Treatment With Rehabilitation in the Future

Although previous studies including our present one show functional benefits by different rehabilitative approaches after SCI (Cote et al., 2017; Torres-Espin et al., 2018; Loy and Bareyre, 2019), rehabilitation alone is limited in promoting significant locomotor recovery (Quel de Oliveira et al., 2017; Tse et al., 2018). Combining rehabilitation with other therapeutic approaches, such as electric stimulation (Field-Fote, 2001; Rejc et al., 2017a), chondroitinase ABC treatment (Wang et al., 2011), or peripheral nerve grafting (Theisen et al., 2017), may be needed to promote greater functional recovery after SCI, especially after chronic SCI. Transplantation of neural stem cells is a promising therapeutic approach for SCI (Nori et al., 2011; Rosenzweig et al., 2018). However, transplantation of neural stem cells alone is more effective at promoting locomotion recovery after subacute SCI but limited after chronic SCI (Tashiro et al., 2016, 2017). Importantly, combining treadmill training with neural stem cell transplantation significantly enhanced therapeutic potential and improved motor function after chronic SCI (Tashiro et al., 2016). We are studying the therapeutic efficacy of neural stem cells derived from human inducible pluripotent stem cells in fine forelimb function after chronic cervical SCI and plan to combine neural stem cell grafting with MMS rehabilitation in the near future. In this study, we first studied the effects of MMS rehabilitation after chronic cervical contusion in nude rats, which are widely used for investigating the safety and therapeutic efficacy of human neural stem cells. Our results show that the major SCI pathophysiological changes in nude rats, such as cavity formation, astroglial responses, and responses of macrophages/microglia, are similar to non-immunodeficient rats. These results are consistent with a previous study showing similar lesion size and cellular inflammatory responses in non-obese diabetic severe combined immunodeficient mice as in other regular mice (Luchetti et al., 2010), supporting the applicability of nude rats for SCI studies, especially for neurotransplantation ones. Importantly, our results show that MMS rehabilitation improves the injury microenvironment and promotes neuroplasticity after chronic SCI, suggesting that rehabilitation could further enhance the therapeutic efficacy of neural stem cell transplantation for chronic SCI.

## Conclusion

Our study has demonstrated that MMS rehabilitation decreases astrogliosis, enhances plasticity in terms of axonal sprouting and synaptic upregulation in the ventral horn caudal to the lesion, and importantly, promotes functional recovery in reaching and grasping after chronic cervical SCI. This study supports the therapeutic potential of task-specific rehabilitation in the recovery of hand function in patients with chronic cervical SCI.

## Data Availability Statement

All datasets generated for this study are included in the article/supplementary material.

## Ethics Statement

The animal study was reviewed and approved by the University of Texas AWC.

## Author Contributions

CG and QC conceived the research idea, designed the experiments, analyzed, interpreted the results, and drafted the manuscript. CG, MC, YZ, and XH performed the experiments and collected the data. CG undertook statistical analysis.

## Conflict of Interest

The authors declare that the research was conducted in the absence of any commercial or financial relationships that could be construed as a potential conflict of interest.
